# Supplementary material for: Combination of Epithelial Growth Factor Receptor Blockers and CDK4/6 Inhibitor for Nasopharyngeal Carcinoma Treatment
Source: Cancers (Basel). 2021 Jun 12;13(12):2954. doi: 10.3390/cancers13122954 (PMC8231497; doi:10.3390/cancers13122954)
Supplement: Supplementary file 1 [file cancers-13-02954-s001.zip › cancers-1235722-supplementary.pdf]

Combination of Epithelial Growth Factor Receptor Blockers and CDK4/6 Inhibitor for Nasopharyngeal Carcinoma Treatment

Hsin-Pai Li, Chen-Yang Huang, Kar-Wai Lui, Yin-Kai Chao, Chun-Nan Yeh, Li-Yu Lee, Yenlin Huang, Tung-Liang Lin, Yung-Chia Kuo, Mei-Yuan Huang, Yi-Ru Lai, Yuan-Ming Yeh, Hsien-Chi Fan, An-Chi Lin, Chia-Hsun Hsieh, Kai-Ping Chang, Chien-Yu Lin, Hung-Ming Wang, Yu-Sun Chang and Cheng-Lung Hsu

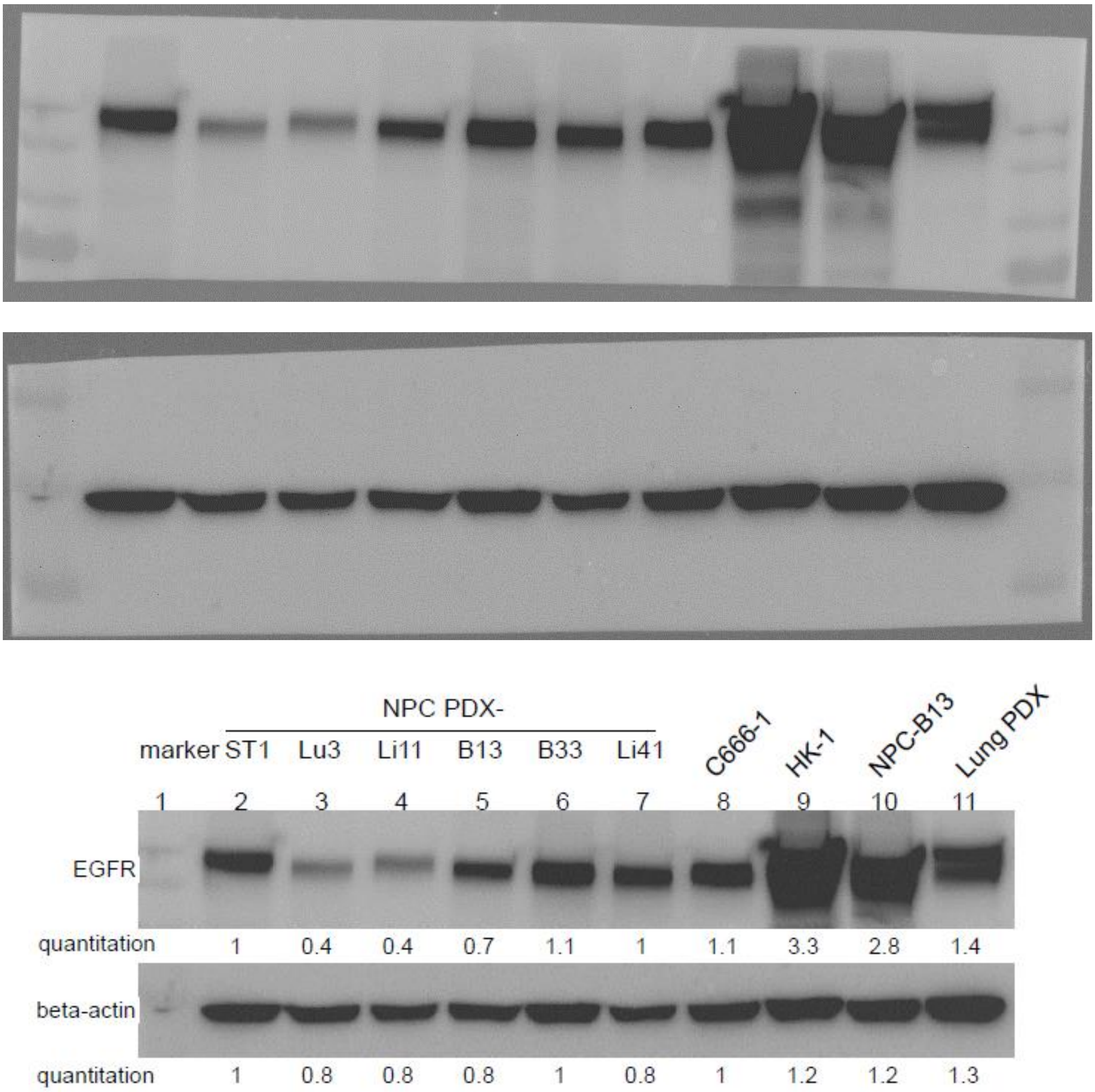

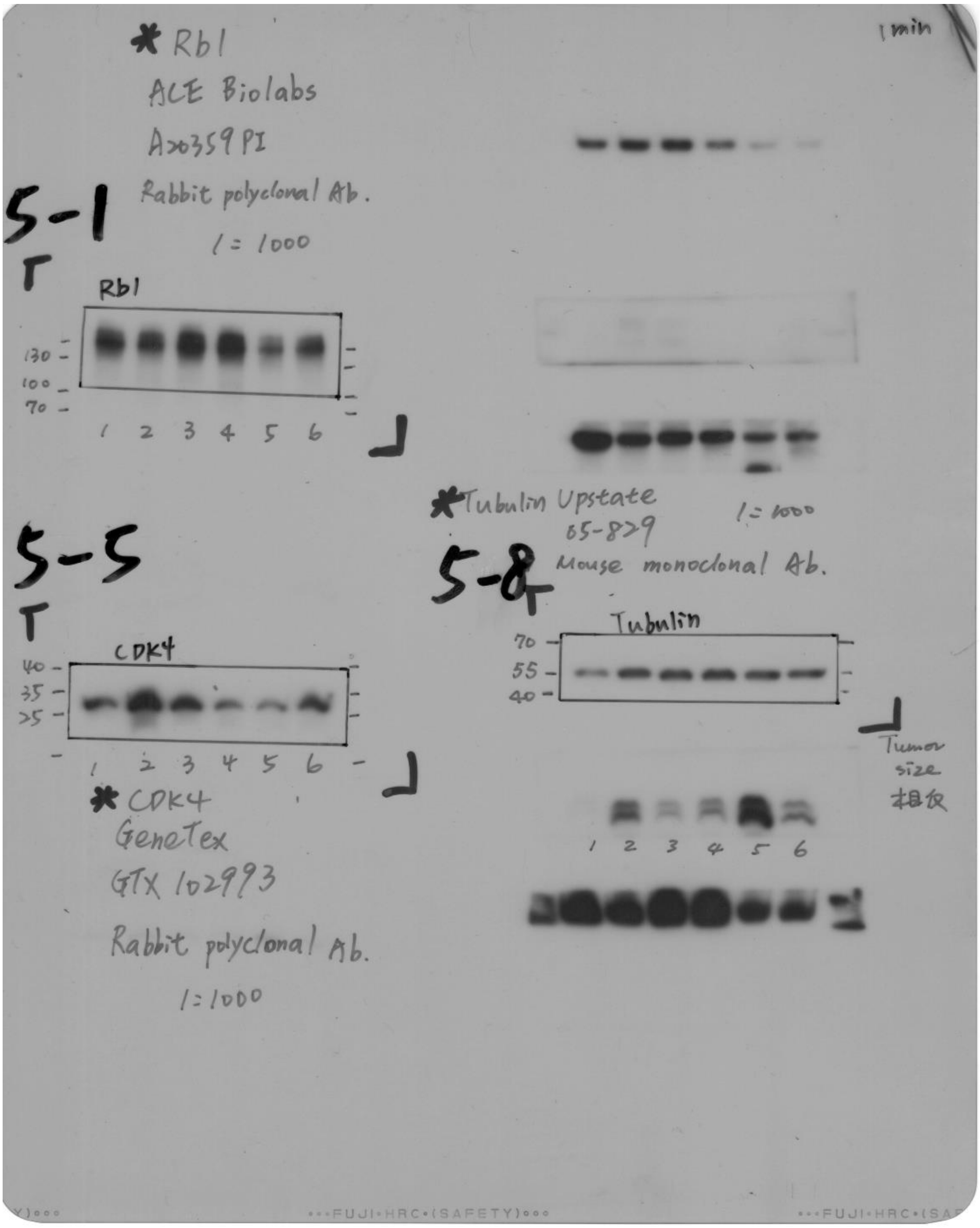
* Rb1
ACE Biolabs
A20359PI
Rabbit polyclonal Ab.
1:1000
1min
5-1
Rb1
130
100
70
1 2 3 4 5 6
5-5
CDK4
40
35
25
1 2 3 4 5 6
* CDK4
GeneTex
GTX 102993
Rabbit polyclonal Ab.
1:1000
* Tubulin Upstate
05-829
1:1000
5-8
Mouse monoclonal Ab.
Tubulin
70
55
40
Tumor size
1 2 3 4 5 6
FUJI•HRC•(SAFETY)

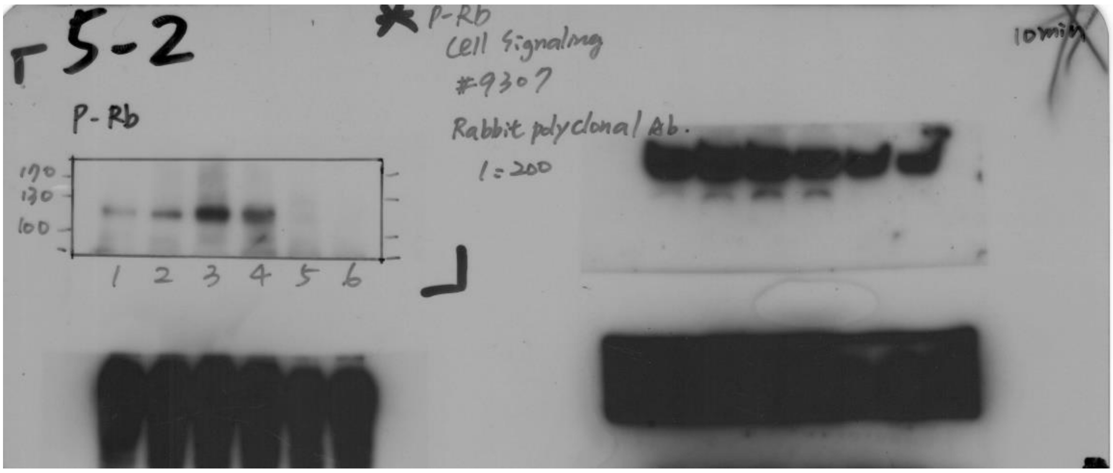
5-2
* P-Rb
Cell Signaling
#9307
Rabbit polyclonal Ab.
1:200
10min
P-Rb
170
130
100
1 2 3 4 5 6

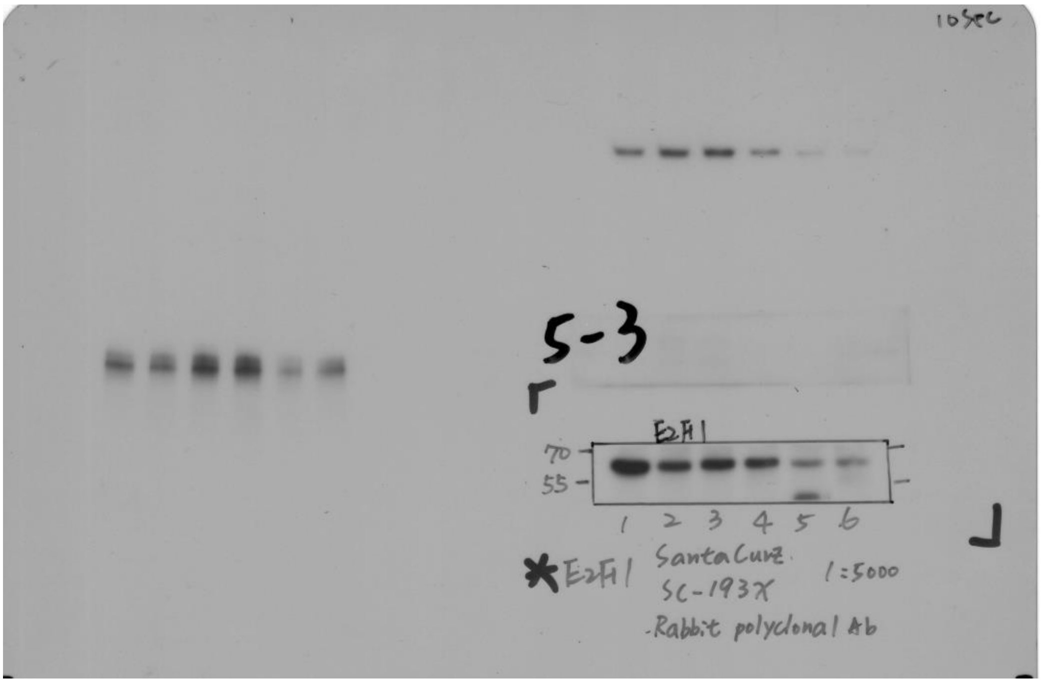
10sec
5-3
E2F1
70
55
1 2 3 4 5 6
*E2F1 SantaCruz SC-193X 1:5000
Rabbit polyclonal Ab

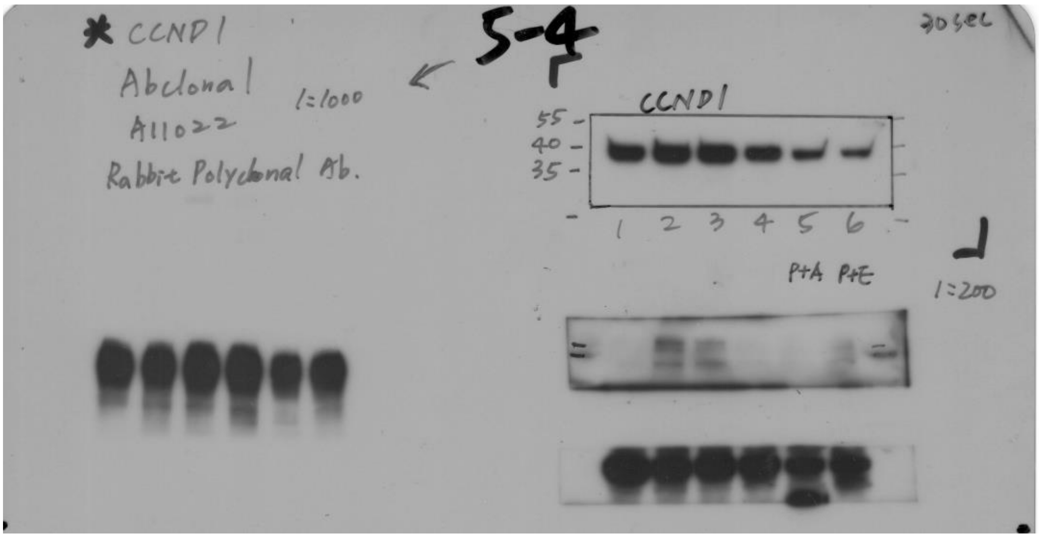
* CCND1
Abclonal 1:1000
A11022
Rabbit Polyclonal Ab.
5-4
30sec
CCND1
55
40
35
1 2 3 4 5 6
P+A P+E
1:200

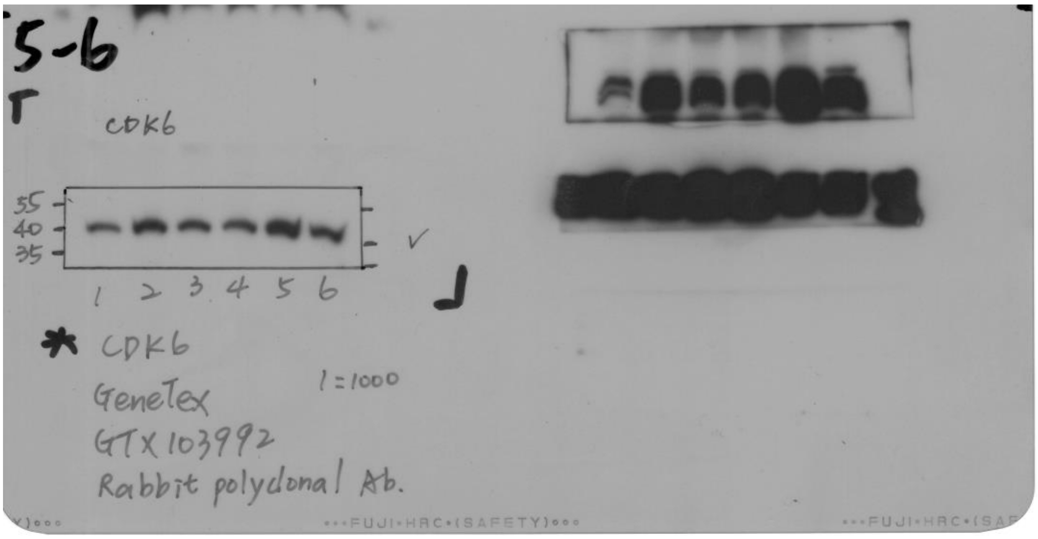
5-6
CDK6
55
40
35
1 2 3 4 5 6
* CDK6
GeneTex 1:1000
GTX103992
Rabbit polyclonal Ab.
FUJI HRC (SAFETY)

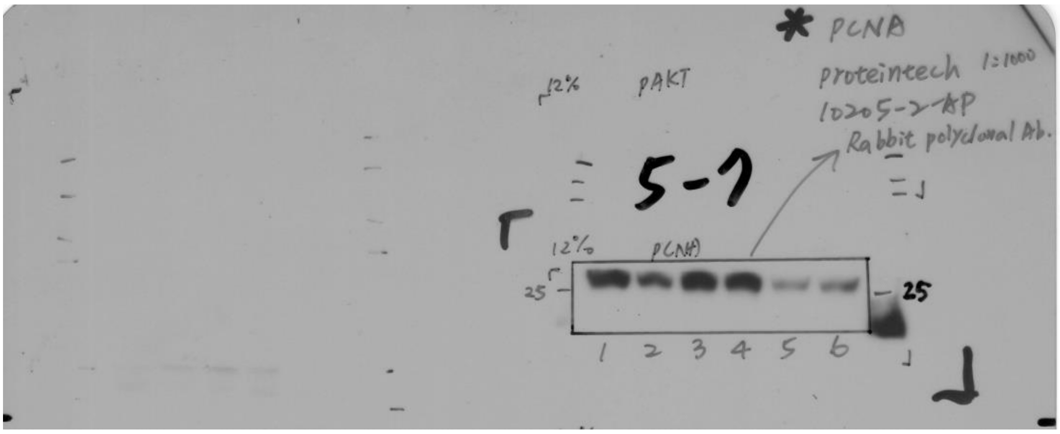
* PCNA
proteintech 1:1000
10205-2-AP
Rabbit polyclonal Ab.
12%
PAKT
5-7
12%
PCNA
25
25
1 2 3 4 5 6

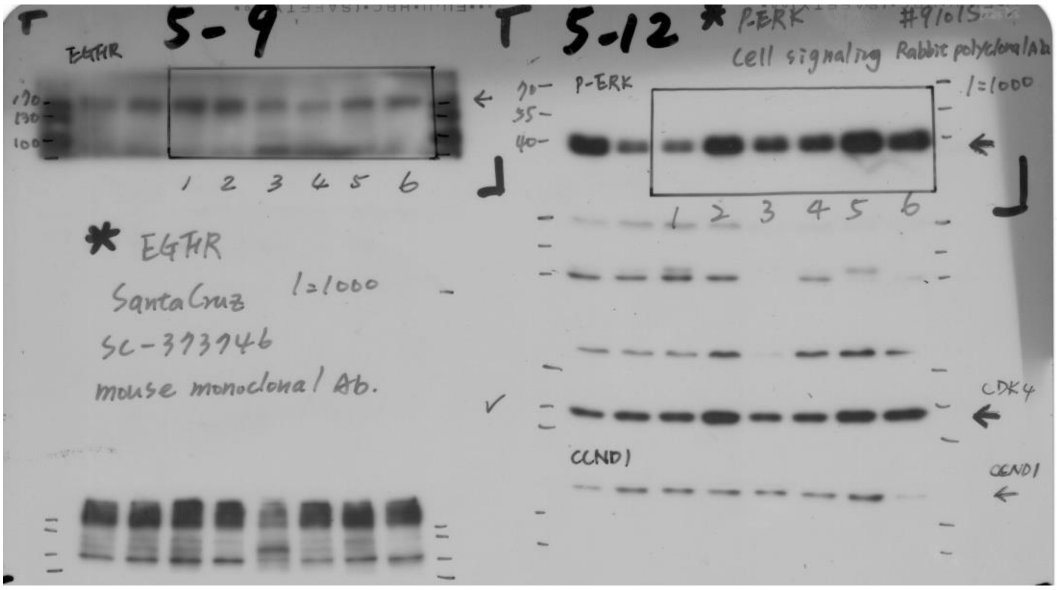
5-9
EGFR
170
130
100
1 2 3 4 5 6
* EGFR
SantaCruz 1:1000
sc-373746
mouse monoclonal Ab.
5-12
* P-ERK
#9101S
Cell signaling Rabbit polyclonal Ab
1:1000
P-ERK
70
35
40
1 2 3 4 5 6
CDK4
CCND1
CCND1

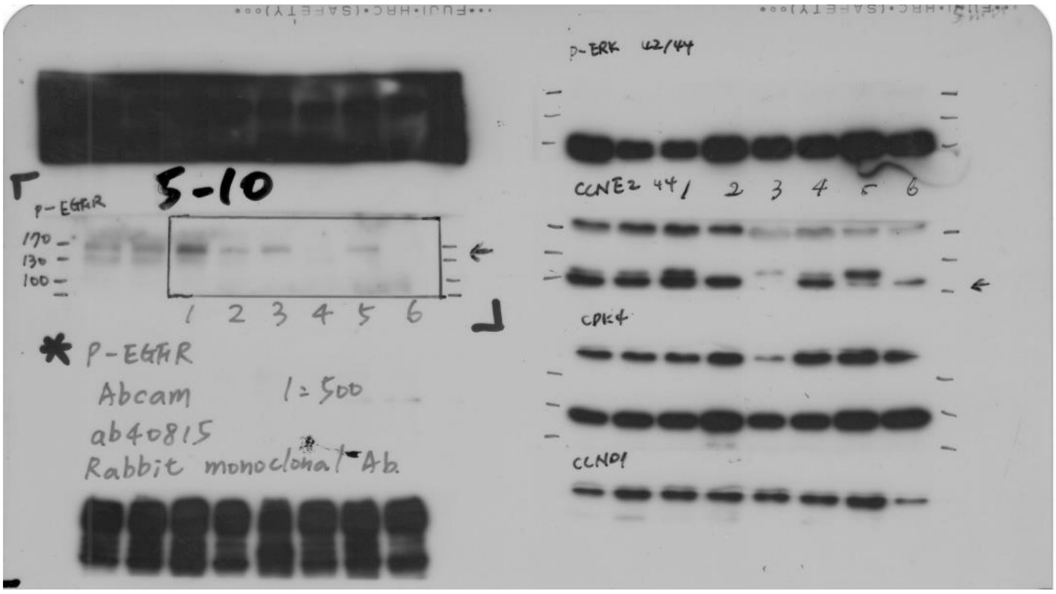
P-ERK 42/44
5-10
P-EGFR
170
130
100
1 2 3 4 5 6
CCNE2 44
1 2 3 4 5 6
* P-EGFR
Abcam 1:500
ab40815
Rabbit monoclonal Ab.
CDK4
CCND1

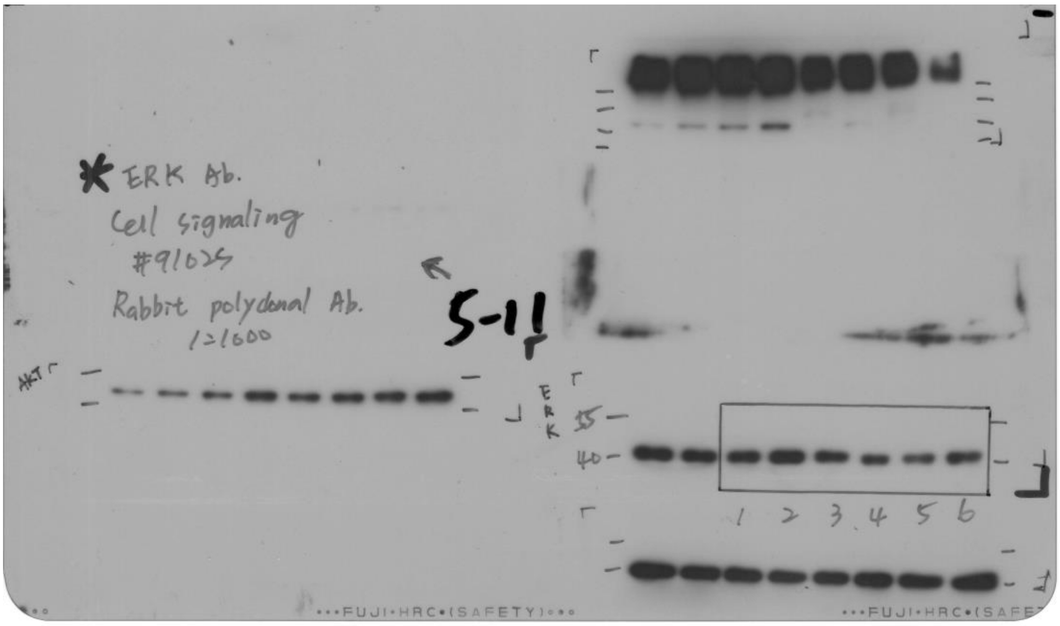
* ERK Ab.
Cell signaling
#9102S
Rabbit polyclonal Ab.
1:1000
5-11
AKT
ERK
55
40
1 2 3 4 5 6
FUJI·HRC·(SAFETY)

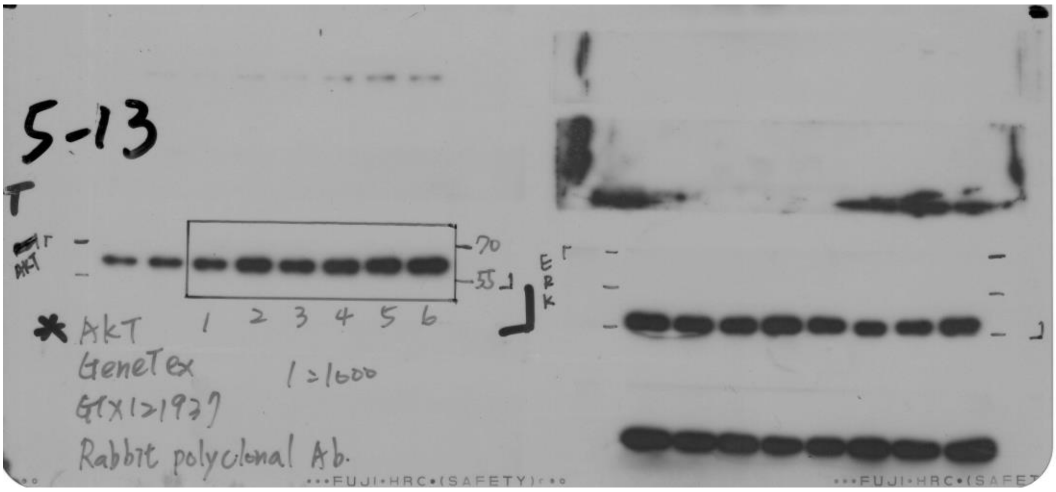
5-13
AKT
70
55
1 2 3 4 5 6
ERK
* AKT
GeneTex
1:1000
GTX121937
Rabbit polyclonal Ab.
FUJI·HRC·(SAFETY)

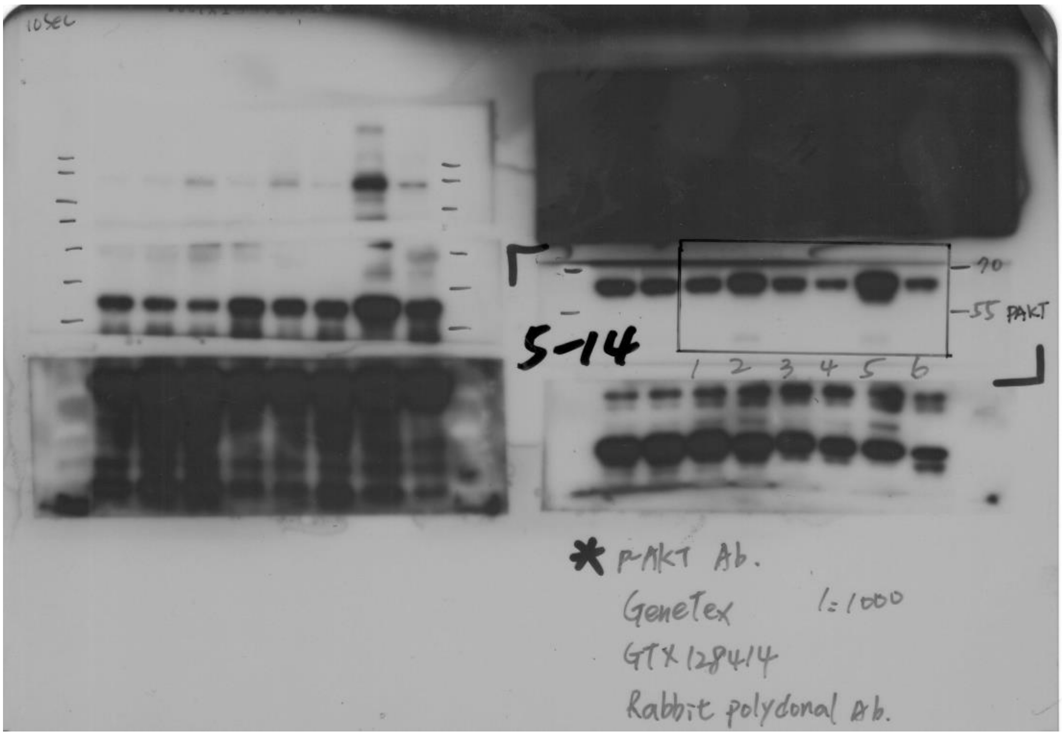
10sec
70
55 PAKT
5-14
1 2 3 4 5 6
* P-AKT Ab.
GeneTex
1:1000
GTX128414
Rabbit polyclonal Ab.

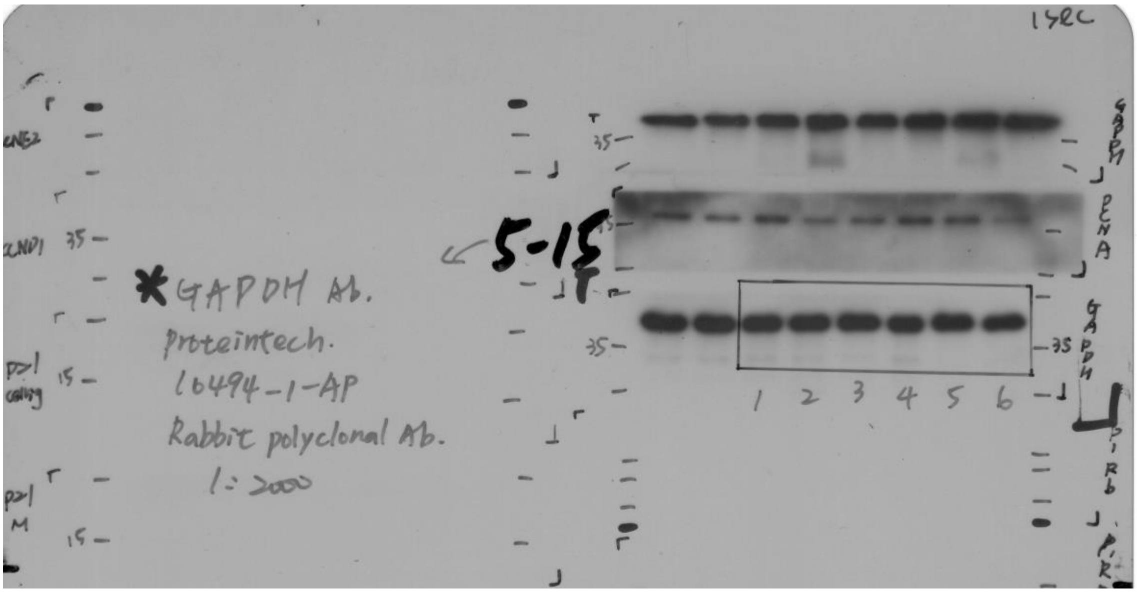

Fig. 5. Western blot analysis EGFR pathway and cell cycle key protein of NPC PDX-B13 tumor with or without EGFRi and/or Palbociclib treatment.

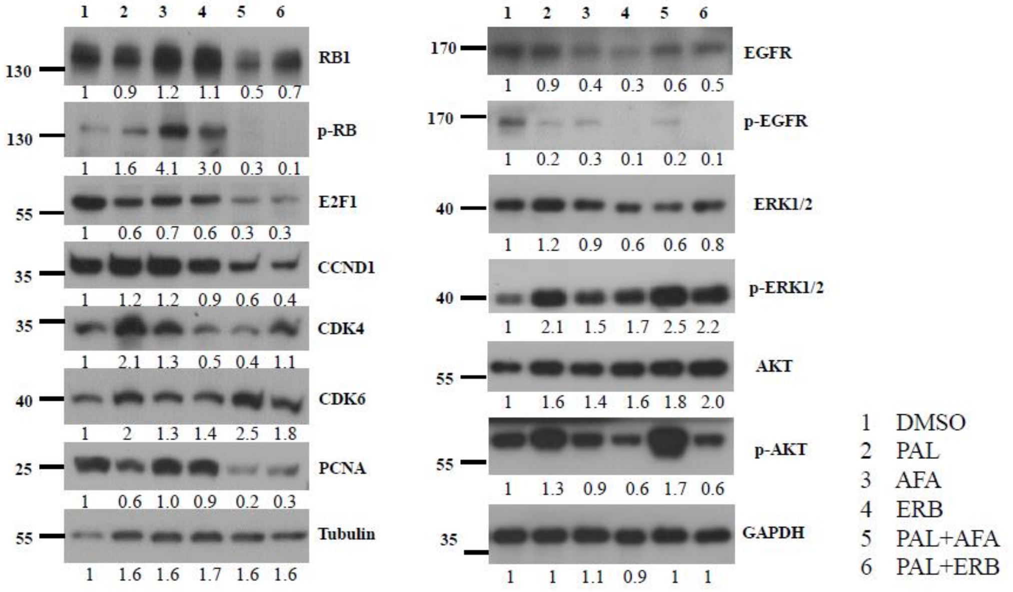

Figure S1. The uncropped western blots.

HPV genotyping by HPV Blot

Fifteen microliters of the resultant amplicons were then hybridized with a HPV Blot membrane. Thirty-eight types of HPV: 6, 11, 16, 18, 26, 31, 32, 33, 35, 37, 39, 42, 43, 44, 45, 51, 52, 53, 54, 55,56, 58, 59, 61, 62, 66, 67, 68, 69, 70, 71(CP8061), 72, 74, 81(CP8304), 82(MM4), 83(MM7), 84(MM8), L1AE5 can be detected in a single reaction as previously described (ref: Role of human papillomavirus status after conization for high-grade cervical intraepithelial neoplasia. Huang HJ, Tung HJ, Yang LY, Chao A, Tang YH, Chou HH, Chang WY, Wu RC, Huang CC, Lin CY, Liao MJ, Chen WC, Lin CT, Chen MY, Huang KG, Wang CJ, Chang TC, Lai CH. Int J Cancer. 2021 Feb 1;148(3):665-672. doi: 10.1002/ijc.33251. Epub 2020 Sep 1.)

(a)

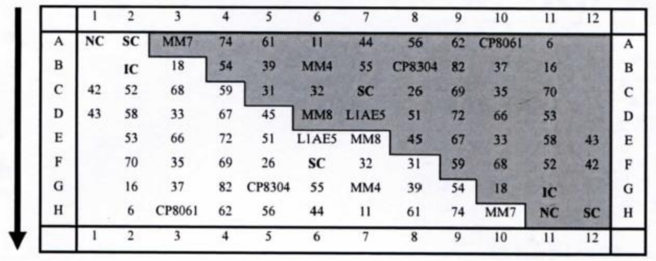

	1	2	3	4	5	6	7	8	9	10	11	12	
A	NC	SC	MM7	74	61	11	44	56	62	CP8061	6		A
B		IC	18	54	39	MM4	55	CP8304	82	37	16		B
C	42	52	68	59	31	32	SC	26	69	35	70		C
D	43	58	33	67	45	MM8	L1AE5	51	72	66	53		D
E		53	66	72	51	L1AE5	MM8	45	67	33	58	43	E
F		70	35	69	26	SC	32	31	59	68	52	42	F
G		16	37	82	CP8304	55	MM4	39	54	18	IC		G
H		6	CP8061	62	56	44	11	61	74	MM7	NC	SC	H
	1	2	3	4	5	6	7	8	9	10	11	12	

(b) 1 2 3 4 5 6 7 8 9 10 11 12

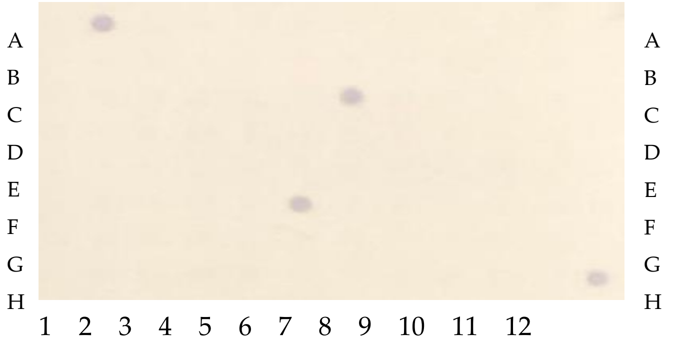

1 2 3 4 5 6 7 8 9 10 11 12

HK-1
=>HPV negative

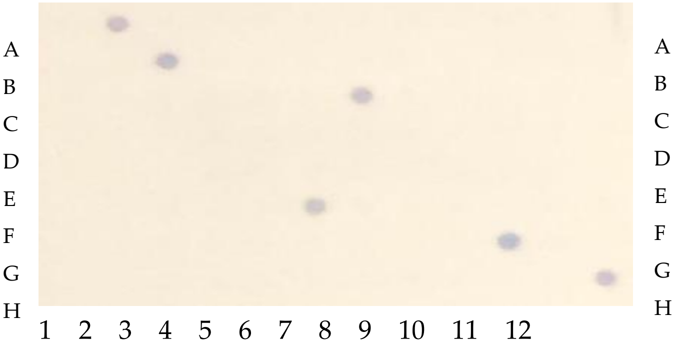

1 2 3 4 5 6 7 8 9 10 11 12

Positive control : Oropharyngeal cancer
=>HPV18

Figure S2. HPV genotyping by HPV Blot. (a) The schematic diagram of HPV blot. (b) The L1 amplicons by using SPF1/GP6+ consensus primers for the detection HPV Blot. L1 amplimers (220bp) was demonstrated on HK1 cell line (HPV negative) and oropharyngeal cancer tissue (HPV18). HPV18 was detected in HPV blot. SC: positive control, NC: negative control.

Table S1. Total 6 metastatic NPC patients' characteristics for PDX.

Patient No.	Age (y/o)	Gender	Status at biopsy/operation			Previous treatment	PDX engraft	Overall survival (months)
			Tissue source	Biopsy(B)/ Excision(E)	EBV DNA load (copies/mL)			
1	42	Male	Soft tissue(ST)	E	4,525	No	Yes	13
3	64	Male	Lung(Lu)	B	87	CCRT	Yes	15
11	52	Male	Liver(Li)	E	3,060	CCRT	Yes	7
13	52	Male	Bone(B)	B	>2,000,000	CT	Yes	5
33	46	Male	Bone(B)	E	13,348	CCRT	Yes	15
41	49	Male	Liver(Li)	B	173	CCRT	Yes	7

1.CCRT: concurrent chemoradiotherapy; CT: chemotherapy.

2.The nomenclature system of our NPC PDX was NPC PDX-(tissue source abbreviation)(patient number). The NPC PDX-ST1 and PDX-B13 were corresponding to NPC PDX-ST and NPC PDX-Bone in reference [J Exp Clin Cancer Res. 2018 Sep 20;37(1):233.]. NPC PDX-B33 and NPC PDX-Li41 were new established NPC PDX lines and were under characterization.

3. NPC-B13 cell line had been passaged for more than 80 passages with EBV episome 40~50 copies/cell *in vitro* but would not form xenograft in NOD/SCID mice.

4.All these PDX lines harbored EBV except NPC PDX-Li41.
